# NMR and GPC Analysis of Alkyd Resins: Influence of Synthesis Method, Vegetable Oil and Polyol Content

**DOI:** 10.3390/polym15091993

**Published:** 2023-04-23

**Authors:** Antonella Hadzich, Santiago Flores, Ashley E. Masucci, Enrique D. Gomez, G. Alexander Groß

**Affiliations:** 1 Instituto de Corrosión y Protección (ICP-PUCP), Pontificia Universidad Católica del Perú (PUCP), Avenida Universitaria 1801, Lima 32, Peru; 2 Department of Chemical Engineering, The Pennsylvania State University, University Park, PA 16802, USA; 3 Department of Materials Science and Engineering, Materials Research Institute, The Pennsylvania State University, University Park, PA 16802, USA; 4 Department of Physical Chemistry and Microreaction Technology, Institute of Chemistry and Biotechnolgy, Technische Universität Ilmenau, Prof.-Schmidt-Str. 26, 98693 Ilmenau, Germany

**Keywords:** alkyd resin, glycerol, GPC, NMR, pentaerythritol, sacha inchi oil

## Abstract

Alkyd resins are oil-based polymers that have been widely used for generations in the surface coating industry and beyond. Characterization of these resins is of high importance to understand the influence of its components on its behavior, compatibility with other resins, and final quality to ensure high durability. Here, NMR spectroscopy and GPC were used for characterizing differences in the chemical structure, molecular distribution, and dispersity between oil-based and fatty acid-based alkyd polymers made from sacha inchi and linseed oils. Sancha inchi (*Plukentia volubilis* L.) is a fruit-bearing plant native to South America and the Caribbean, and has a rich unsaturated fatty acid content. The effect of vegetable oil and polyol selection on the synthesis of alkyd resins for coating applications was analyzed. The influence of two different synthesis methods, monoglyceride and fatty acid processes, was also compared. Important structural differences were observed using NMR: one-dimensional spectra revealed the degree of unsaturated fatty acid chains along the polyester backbone, whereas, 2D NMR experiments facilitated chemical shift assignments of all signals. GPC analysis suggested that alkyd resins with homogeneous and high molecular weights can be obtained with the fatty acid process, and that resins containing pentaerythritol may have uniform chain lengths.

## 1. Introduction

Alkyd resins are polyesters modified using vegetable oils [1]. Conjugated carbon double-bonds from fatty acid chains, preferably catalyzed by metallic driers, afford the polymer the ability to generate radical autoxidation reactions [2], which facilitate its cross-linking and the formation of a dry film. This inherent characteristic gives alkyd resins the advantage of being single component coatings and, due to their oily content, they can better tolerate the presence of rust on the surface. In addition to being easy to apply, these vegetable oil-based coatings are cheap and have been successfully used in a variety of applications, including industrial, decorative, architectural or artistic coatings [3,4,5,6]. In recent years, they are being explored for self-healing applications to ensure the long-term durability of protective coatings [7,8]. Alkyd resins also have a great compatibility with other resins, and have been blended with other polymers to improve their performance [4].

The wide range of reagents that can be used for the synthesis of alkyd resins increase their versatility, and their structures and film properties can be tuned by their components [9]. Vegetable oils as fatty acid sources, polycarboxylic acids, such as phthalic anhydride (PA), and polyols, such as glycerol (GC) or pentaerythritol (PE), are commonly used for alkyd resin production [10]. Except for phthalic anhydride, alkyd polymers are based on natural resources and can be classified as biologically degradable materials [11]. Recent work has demonstrated the possibility to reduce non-renewable component and incorporate the use of waste products, such as polyethylene terephthalate (PET) from postconsumer bottles [12], to manufacture this type of resins.

We examined alkyd resins that contain different glycerol (GC): pentaerythritol (PE) weight ratios. Glycerol was the first polyol to be used in the production of alkyd resins. GC is a cheap and easily available renewable raw material, which contains two primary and one secondary hydroxyl groups [13]. On the other hand, pentaerythritol has the highest functionality, four hydroxyl groups per molecule, which causes the formation of more branched and higher molar mass alkyd resins [14]. It has been used as an alternative to glycerol in alkyd formulations to impart high viscosity, greater hardness, improved hydrolytic resistance, color stability, thermal stability, and external durability [15,16,17]. Due to the high reactivity of PE and its ability to increase branching [15], that could cause gelation or loss of resin, we examined different proportions of GC and PE in the alkyd resin synthesis.

Alkyd resins can be classified in accordance with their oil length or weight percentage of oil: long (>55%), medium (45–55%) and short oil (<45%) [3,18]. Long oil alkyd resins are used in clear lacquers for decorative or artistic paints, whereas medium oil alkyd resins have a wider industrial application, being used as primers, maintenance paints and metal finishes. Short oil alkyd resins are used in baking primers and enamels. [18]. On the other hand, the iodine value of oils or degree of unsaturation, determines its classification as drying, semi-drying or non-drying. Long and medium oil alkyd resins are frequently produced from semi-drying or drying oils [3,16]. Alkyd resins synthesized with non-drying oils are used as plasticizing agents [18]. The alkyd resins analyzed in this study have a medium chain length.

The properties of alkyd resins strongly depend on the type of vegetable oil precursor [17]. The high content of polyunsaturated fatty acids have turned linseed oil into one of the most used oil resource for alkyd manufacturing [19,20]. However, in recent years, non-traditional and new feedstocks of vegetable oils are being investigated for polymeric resin synthesis [21]. This work reports the nuclear magnetic resonance (NMR) and gel permeation chromatography (GPC) characterization of the new alkyd polymers made of sacha inchi oil (*Plukentia volubilis* L.) and linseed oil (*Linum usitatissimum* L.). Linseed oil was used for comparison purposes. Sacha inchi oil, of Peruvian origin, was selected due to its rich unsaturated fatty acids content [22]. Its similarity to the composition of linseed oil [23] makes it a potential raw material for new alkyd coatings. Recent work has shown that sacha inchi oil is a new and effective feedstock for alkyd resins synthesis in the surface coating industry, either as pure oil [17,24,25,26], through its fatty acids [16] or as part of an oil mixture [27,28]. Its effectiveness has also been proven as a raw material for the creation of artistic products [6]. Currently, sacha inchi seeds are used for oils, cakes and protein meals, and are highly used in the cosmetic, food and medicine industry [28].

NMR analysis has been extensively used for elucidating the chemical composition of alkyd resins [29,30,31,32,33,34]. Moreover, the use of 2D NMR spectroscopy has been successfully applied to distinguish between multiple components of broad and/or featureless peaks in 1D NMR spectra [29]. On the other hand, GPC has been used to evaluate the molecular weight and dispersity of alkyd resins [21,30,34,35,36]. These properties are of crucial importance, as they can affect the quality of alkyd resins [35,37].

This work examines the structural differences between the new alkyd resins obtained from sacha inchi and linseed oils. Two different synthesis methods were used: (A) The monoglyceride process. (B) The fatty acid process. Both processes can be performed with the same starting materials, but fatty acids from the vegetable source must be first extracted in the fatty acid process, which makes it costly [16,38]. Due to the high reactivity of fatty acids, the synthesis time is shorter, however greater control is required to avoid gelation of the resin [16]. In this study, alkyd resins were manufactured with both methods, and with precursors that have similar fatty acid content, but that vary in content of polyols or that contain a mixture of polyols, and the same polyacid.

## 2. Experimental Section

### 2.1. Materials

All samples were provided by the Instituto de Corrosión y Protección, Pontificia Universidad Católica del Perú. All alkyd resins contain different glycerol (GC): pentaerythritol (PE) weight ratios. Linseed oil was used for comparison purposes. “A” alkyd samples were synthesized using the monoglyceride method, whereas “FA” alkyd samples were synthesized using the fatty acid process. Fatty acid fractions were obtained from oils using a base-catalyzed transesterification reaction [16]. (F)AS were prepared with sacha inchi oil/fatty acid, while (F)AL were prepared with linseed oil/fatty acid. Alkyd resins were identified with numbers from 1 to 3, depending on their content of polyol: (F)AS1 and (F)AL1 contain 1:0 GC:PE; (F)AS2 and (F)AL2 contain 0.5:0.5 GC:PE; (F)AS3 and (F)AL3 contain 0.2:0.8 GC:PE (Table 1). All samples were prepared with the same amount of phthalic anhydride and oil/fatty acid fraction [16,17].

### 2.2. NMR Spectroscopy

NMR analysis was performed in the Department of Physical Chemistry and Microreaction Technology at the Technische Universität Ilmenau (Ilmenau, Germany) with a Bruker Avance 300 spectrometer. Samples were dissolved with deuterated chloroform (10 wt.%). Data analysis was performed using the MestReNova v12.0.4-22023 software.

### 2.3. Gel Permeation Chromatography

Gel Permeation Chromatography (GPC) was performed in the Department of Chemical Engineering at The Pennsylvania State University (USA). Molecular weights and molecular weight distribution (Mw/Mn) were determined with the 1260 Agilent Technologies gel permeation chromatograph (GPC) equipped with a refractive index detector (RID), a viscometer (VS), a light scattering detector (LS) and a multiwavelength detector (MWD), using an Agilent PLgel-MIXED-LC column. Chlorobenzene was used as the mobile phase at 0.5 mL/min and the sample concentration was ~30 mg/mL. Universal GPC calibration curve was prepared with low polydispersed linear polystyrene standards (Agilent EasiVial PS-M, Agilent, Santa Clara, CA, USA).

## 3. Results and Discussion

### 3.1. 1D ^1^H-NMR Spectra

**Figure 1:**
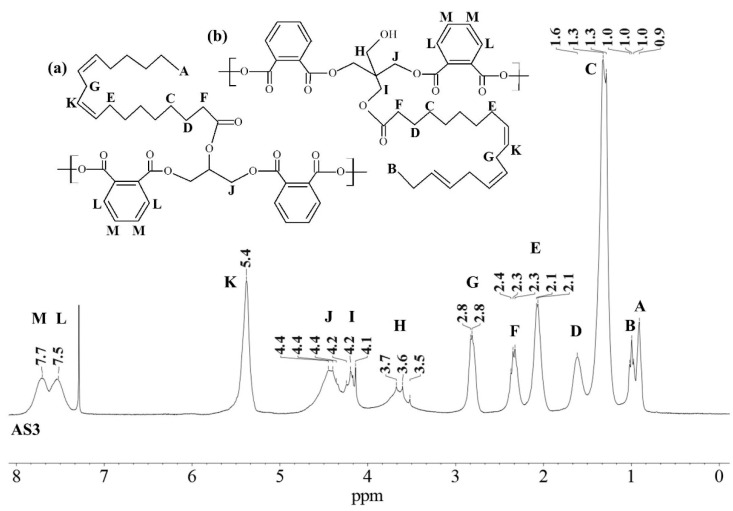
^1^H-NMR spectra of a sacha inchi-based alkyd resin, AS3 at 300 MHz in deuterated chloroform. Polymer structure comparison: (**a**) glycerol-based alkyd resin with linoleic (C18:2) fatty acid chain; (**b**) pentaerythritol-based alkyd resin with linolenic (C18:3) fatty acid chain.

Similar ^1^H NMR (300 MHz, chloroform-d) spectra were obtained for all alkyd resins. Figure 1 shows the ^1^H NMR spectra that contains thirteen signals typical of an alkyd resin classified with letters (A) to (M), starting from the high field region. Possible location of protons corresponding to each peak in alkyd resin structures (representing possible repeating units) is also identified in Figure 1. The unmarked peak at δ 7.28 ppm corresponds to chloroform-d. The assignment of ^1^H-NMR chemical shifts in FAS3, AS3, AL3, AS1 and AL1 alkyd resins are summarized in Table 2. For the NMR resonance description, the “NMR Guidelines for ACS Journals” standard was used. For example, AS3 can be summarized as follows: δ 7.71 (singlet (s), 2H), δ7.54 (s, 2H), δ 5.38 (s), δ 4.70–4.31 (multiplet (m)), δ 4.29–4.08 (m), δ 3.83–3.47 (m), δ 2.96–2.70 (m), δ 2.43–2.23 (m), δ 2.19–1.96 (m), δ 1.61 (s), δ 1.30 (doublet), δ 1.07–0.95 (m), δ 0.95–0.84 (m).

The low field region of the spectrum at δ = 6.5–8.0 ppm aromatic ring protons, peaks (L) and (M), from the phthalic ester moieties. The resonance at about δ = 5.4 ppm, peak (K), was assigned to the vinylic protons. Intermediate signals δ = 3.4–4.8 ppm, composed of peaks (H), (I) and (J), correspond to the protons assigned to the polyol(s) CH_2_ groups. The resonance at about δ = 2.8 ppm, peak (G), was assigned to aliphatic CH_2_ groups shielded by two neighboring vinyl groups. Remaining peaks in the aliphatic region δ = 0–2.6 ppm correspond to sp^3^ C bound protons assigned to the fatty acid chains, as shown in Table 2. The proton resonance assignment of all peaks is summarized in Table 2 and they align with data in the literature [29,32,33,39]. Previous studies of alkyd resins were reported by Boruah et al. [21], Spyros [29], Chiplunkar and Pratap [31], Rämänen and Maunu [33], and Glenn et al. [34].

**Figure 2:**
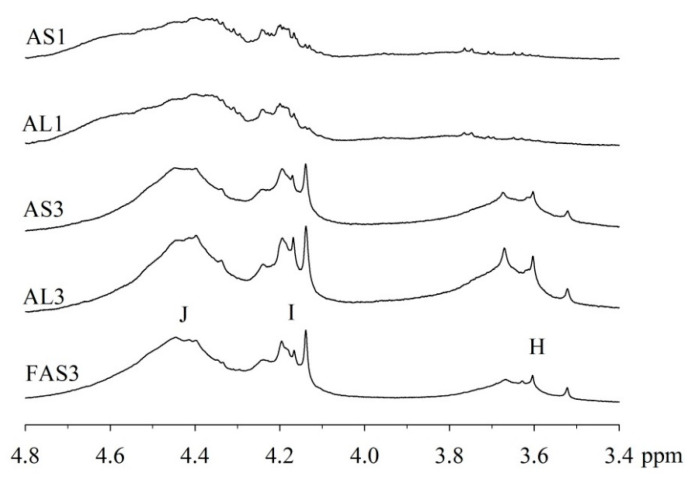
Expanded ^1^H-NMR spectra of the polyol region (δ 3.4–4.8 ppm) of alkyd resins at 300 MHz in chloroform-d.

An expansion of the ^1^H spectra polyol region of the alkyd resins is shown in Figure 2. Through a visual comparison of all the spectra, a clear difference can be seen between alkyds prepared with glycerol (e.g., AS1, AL1) and those prepared with pentaerythritol (e.g., AS3, AL3, FAS3). As specified in Table 2, signal (H) correspond to methylene protons of the CH_2_OH groups. The degree of branching depends on the esterification degree of the polyol unit [33]. However, PE has a higher degree of OH-functionality than GC. Because of the steric differences, there is a higher probability that more unreacted CH_2_-OH groups remain in the PE structure in comparison to GC. This assumption is supported by the observation that the intensity of peak (H) is more intense in case of the PE alkyds (AL3, AS3, FAS3) compared to the GC alkyds (AL1, AS1). Peak (I), assigned to the esterified CH_2_-O- moieties of the PE-containing alkyd resins, has higher signal intensity because of the greater amounts of PE in the batch. On the other hand, peak (J)’s intensity is comparable for all samples, because the same amount of phthalic anhydride was used for the synthesis of all alkyd resins variants.

The integrated peak area (M) at δ 7.71 ppm, corresponding to two protons attached to the aromatic ring of the phthalic anhydride structure, was used for integral normalization of the full spectrum. The comparison of different alkyd resin samples revealed different relative proportions of polyol and fatty acid chain proton integrals. We assume that that all polymer samples had the same amount of phthalic anhydride, because the same relative amount of phthalic acid was used for the preparation of each batch. Peaks (H), (I) and (J) from the polyol region were chosen to make comparisons between the different samples. Peaks (A) and (B) were also selected, since they are representative signals of the linoleic and linolenic fatty acids, or the main components of vegetable oils used for alkyd synthesis. Other peaks were not taken into account because they might be present in a similar proportion in every possible structure of a repeating unit of an alkyd polymer.

**Figure 3:**
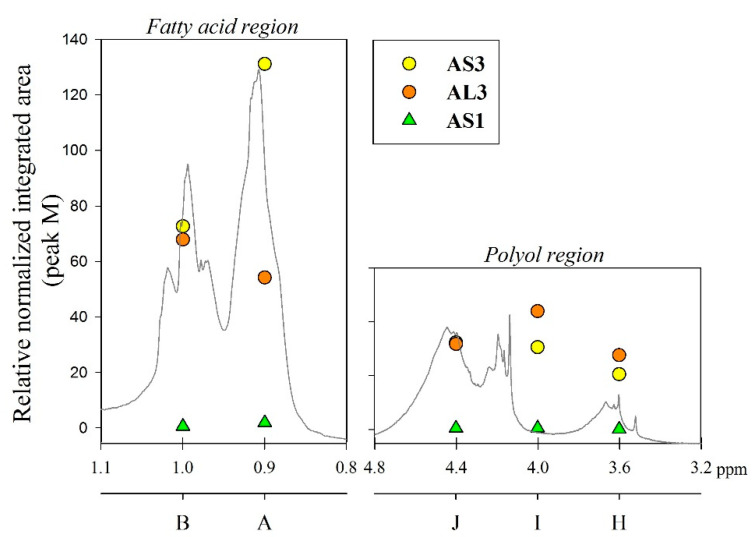
Relative normalized integrated areas of the signals corresponding to polyol (peaks H, I and J from Figure 1) and fatty acid chain protons (peaks A and B from Figure 1) in alkyd resins.

Relative normalized integrated areas of the characteristic signals corresponding to the polyol and fatty acid chain protons are illustrated in Figure 3. Relative proportions of the polyol protons corroborated the visual comparison made with the ^1^H-NMR spectra. Protons in the polyol region are in greater proportion in PE-based alkyd resins. It was also noted that PE-based resins contain more fatty acid chains, due to the highest functionality of pentaerythritol. In the high field region, sacha inchi-based resins have more terminal methyl groups found in a linoleic acid (omega-6) structure (peak (A) from Figure 1) than linseed-based resins (Figure 3). It has been reported that sacha inchi has a similar omega-3 fatty acid composition to linseed oil; though, its omega-6 fatty acid content is higher [22]. A higher degree of unsaturation, i.e., large quantity of double bonds, could enhance resin drying properties and, therefore, the hardness of cured resins. The development of extensive cross-linking would lead to the obtainment of a better quality product [29,40].

### 3.2. 1D ^13^C-NMR Spectra

**Figure 4:**
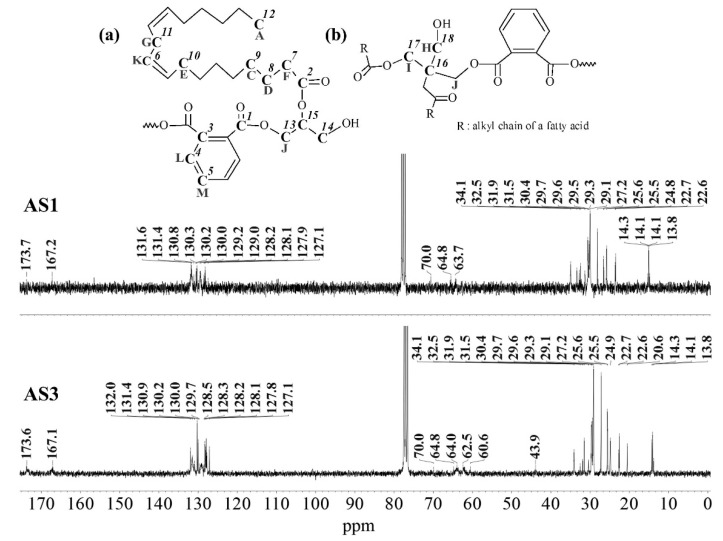
^13^C-NMR spectra of alkyd resins AS1 and AS3 at 75 MHz in deuterated chloroform: (**a**) glycerol-based alkyd resin unit with linoleic (C18:2) fatty acid chain; (**b**) pentaerythritol-based alkyd resin unit.

**Figure 5:**
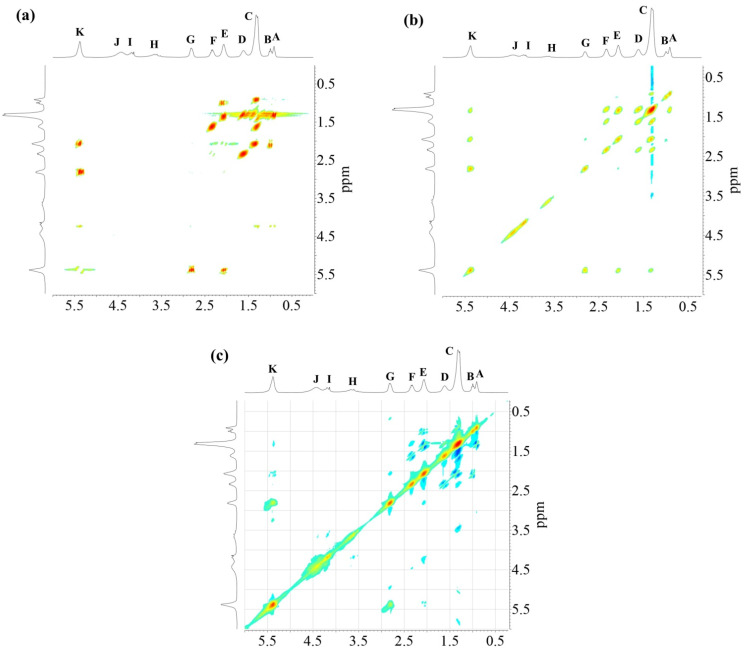
^1^H -^1^H NMR-2D spectra of alkyd resin AS3: (**a**) COSY-90; (**b**) TOCSY; (**c**) ROESY.

**Figure 6:**
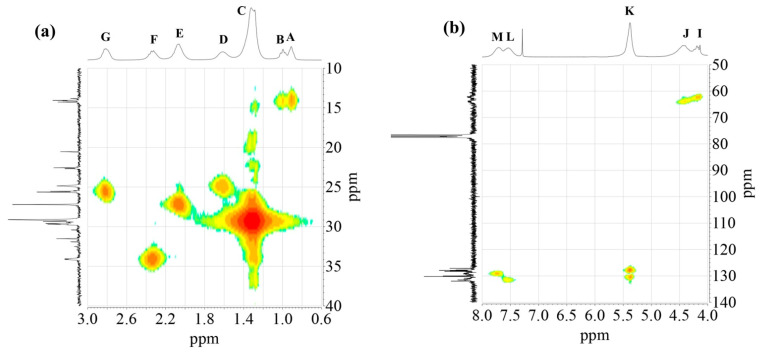
^1^H-^13^C HMQC spectra of alkyd resin AS3: (**a**) high field region; (**b**) low field region.

^13^C-NMR spectra of the alkyd resins were recorded and analyzed accordingly. Representative spectra of resins AS1 and AS3, and their resonance/structure assignment are shown in Figure 4. The 2D-heteronuclear correlation spectra (see Figure 5 and Figure 6) were considered during the assignment of the resonances. Carbonyl groups of phthalic and fatty acid esters (O=C-HC=CH-C=O) were observed at δ = 173.6 ppm (C1) and δ = 167.1 ppm (C2), respectively [12,34]. The region from δ = 127.1 ppm to δ = 131.9 ppm contained aromatic carbons, C3 to C5, and vinyl carbons of unsaturated fatty acid chains (C6) [12,29,34].

Signals of terminal methyl carbons -CH_3_, and internal methylene carbons -(CH_2_)n- from the fatty acid chains, C7 to C12, appeared in the high field region, between δ = 10 and 40 ppm [12,29,33]. Carbon atoms, directly bonded to an oxygen atom in polyalcohol moieties, were perceived in the δ = 40–70 ppm region [34]. Glycerol-based resin AS1 presented three characteristic peaks: C13 at 64.8 ppm, C14 at 63.7–64.0 ppm and C15 at 70.0 ppm [29]. Whereas, resin AS3 prepared with pentaerythritol and glycerol showed the presence of a low intensity peak, corresponding to the quaternary carbon of pentaerythritol (C16) [29]. Moreover, pure glycerol peaks were observed, along with signals coming from the pentaerythritol-based resin units, such as peaks at δ = 62.5 ppm (C17), and δ = 60.6 ppm (C18).

### 3.3. 2D NMR Spectra

Sample AS3 was analyzed using different 2D NMR experiments in order to investigate the structure in greater detail. The ^1^H-^1^H COSY-90 (Figure 5a), ^1^H-^1^H TOCSY (Figure 5b), and ^1^H-^1^H ROESY (Figure 5c) correlation spectra were recorded. The analysis revealed clearly the coupling of peak (K) with peaks (E) and (G), located in the fatty acid chain (Figure 5a). Moreover, coupling of proton peaks (C), (D) and (F) were confirmed. It was corroborated that protons from terminal methyl groups of linoleic acid chain correspond to peak (A), as it has a strong coupling with protons from peak (C). Thus, peak (B), protons from terminal methyl groups of linolenic acid chain, showed a crosspeak with signal (E) in the COSY and ROESY measurements. It should be noted that methylene protons of peak (E) are also close to those of peak (C). The TOCSY-2D spectra (Figure 5b) additionally confirmed that protons of unsaturated carbons identified as peak (K), correlates with protons from internal methylene groups of the aliphatic chains (peak (C)).

The 2D ^1^H-^13^C HMQC spectra (Figure 6) were also acquired for AS3 sample. Figure 6a shows the expanded spectra containing identified ^1^H NMR peaks (A) to (G) (Figure 1), located in the δ 0.6 to 3.0 ppm region, and carbons C7 to C12 that appeared from δ 10 to 40 ppm in the ^13^C NMR spectra (Figure 4). Proton peaks (A) and (B) showed the expected connectivity with carbon peaks around δ 13.8–14.3 ppm, C12 identified in Figure 4, which represent terminal methyl groups on fatty acid chains.

Heteronuclear coupling was used for the identification of the different ^13^C NMR resonances to the methylene protons of the fatty acid chains. Here, peak (C) has a cross correlation with the carbon signals around δ = 29.1–29.7 ppm (identified in Figure 4 as carbon C9). The correlation of the proton peak at δ = 1.61ppm (peak (D)) with a carbon C8 peak at δ = 24.9 ppm, is attributed to the methylene groups located next to -CH_2_COO- group. Proton peak (E) has a cross correlation with carbon C10 peak at δ = 34.1 ppm. Other cross correlations observed in this spectrum originated from coupling between the couples proton peak (F)-carbon C7 peak, and proton peak (G)-carbon C11 peak, that appeared at δ 34.2 ppm and δ 25.5–25.6 ppm, respectively.

The ^1^H-^13^C HMQC spectra of the polyol and aromatic regions of an alkyd resin are presented in Figure 6b. Detected signals of the expanded spectra were found in the range of δ 4.0–8.0 ppm in the ^1^H NMR experiment, which included peaks (I) to (M) (Figure 1). It should be noted that peak (H) showed no clear correlation with a carbon peak. Carbon atoms located in the polyalcohol moiety near aromatic rings (proton peak (J) [32]) appear at higher chemical shifts (e.g., δ 64 ppm) than those closer to fatty acid chains (proton peak (I)). Unsaturated carbons of the fatty acid chains, identified as C6 in Figure 4 and assigned to the proton signal (K), exhibited chemical shifts around δ = 127 and δ = 130 ppm. Aromatic carbons attached to protons (L) and (M) (Figure 1) appeared at δ = 131 and δ = 129 ppm, respectively, which was specified using Spyros [29] as well.

### 3.4. GPC Analysis

The molecular weight averages, ${\bar{M}}_{w}$ (weight-average molar mass) and ${\bar{M}}_{n}$ (number-average molar mass), and dispersity (Đ) of alkyds are summarized in Table 3. With regard to Sacha inchi-based resins, ${\bar{M}}_{n}$ and ${\bar{M}}_{w}$ varied in the order (F)AS3 > (F)AS2 > (F)AS1, whereas, linseed-based resins did not show a specific trend. Linseed oil-based resins had the lowest ${\bar{M}}_{w}$ values, which tend to decrease as the PE content increase. Linseed fatty acid-based alkyds prepared with pure glycerol presented the highest molecular weight averages values.

**Figure 7:**
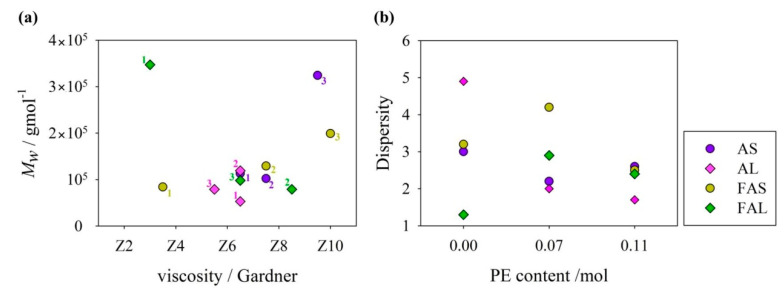
GPC results of alkyd resins: (**a**) Molecular weight average molar mass versus Gardner viscosity (numbers from 1 to 3 represent the polyol rate as detailed in Table 1). (**b**) Dispersity versus molar content of pentaerythritol in alkyd composition.

The molecular weight distribution was correlated with the viscosity of resins, as previously reported by our research group [16,17]. As seen in Figure 7a, oil-based resins that had Gardner viscosities in the range between Z5 and Z8 presented a ${\bar{M}}_{w}$ lower than 1.4 × 10^5^ g/mol. High molecular weights have been related to high degrees of cross-linking [41]. On the other hand, fatty acid-based resins, despite having a greater viscosity range from Z3 to Z10, presented higher and homogeneous degrees of cross-linking. It is important to remark that fatty acid-based resins had a ${\bar{M}}_{w}$ near or even higher than 10^5^ g/mol.

Results from Table 3 clearly indicate that the size distribution of almost all resins is broad (Đ ≥ 2). There are some GPC studies of alkyd resins prepared only with glycerol as the main polyalcohol, which report the following dispersity values: (i) *Jatropha Curcas* oil-based alkyd, Đ = 1.15 [21]; (ii) *Ricinodendron heudelotii* oil-based alkyd, Đ = 1.5 [30]; (iii) *Salvia Hispanica* L. (Chia) oil-based alkyds, Đ = 1.2–4.3 [34]; (iv) rubber seed oil-based alkyd, Đ = 1.6 [35]. However, the oil chain length of these alkyd resins is different, as well as the saturation index of the oils used.

**Figure 8:**
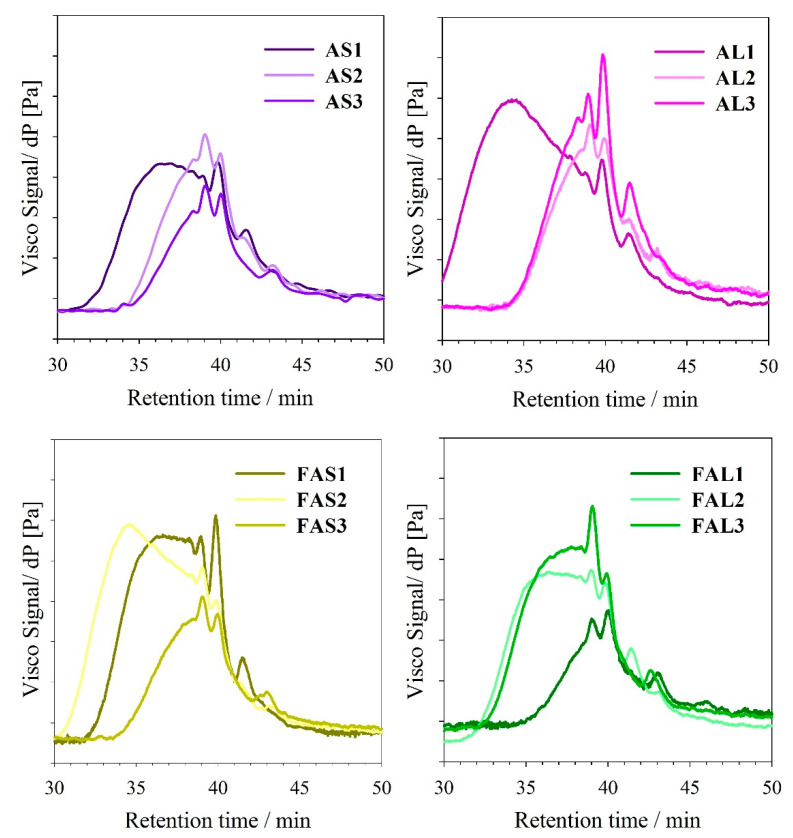
GPC chromatograms of alkyd resins.

In general, dispersity values of oil-based resins decreased as the PE content increased (Figure 7b). GPC traces of PE-based alkyds tend to approximate a symmetric and narrow distribution, which would imply the formation of a more branched polymer (Figure 8) [42]. Đ of linseed oil-based alkyd resins is more dependent on the PE content than the alkyd resins from sacha inchi oil. Oil-based alkyd resins prepared with pure glycerol presented higher dispersity values, which could be attributed to side reactions, such as glycerol oligomerization [43]. In the side reactions, not only glycerol monoesters but also diglycerol and diglycerol monoesters (sub products) react in the polycondensation process, possibly creating polymers of different chain lengths.

Linseed fatty acid-based alkyds had dispersity below 3, while SIFA-based resins have Đ values over 2.5. FAL1 sample had the lowest dispersity. On the other hand, samples with a higher content of PE (e.g., FAS3, FAL3) had similar Đ, despite being synthesized with different fatty acid sources. Đ trend is different from that observed in the case of oil-based alkyd resins, possibly because of the differences in reaction times or minimal differences in the preparation of the fatty acids during the synthesis process. However, the alkyds prepared with the highest PE content had the lowest variation in the dispersity values.

## 4. Conclusions

Alkyd resins were successfully characterized using NMR spectroscopy and GPC. Structural differences between polymers prepared with dissimilar polyols, glycerol and pentaerythritol, and diverse plant sources, sacha inchi and linseed oils, were found using the NMR spectroscopy. In the high field region of 1D NMR spectra, the peaks corresponding to terminal methyl groups, found in the δ = 0.8–1.1 ppm region, allowed us to identify the approximate amount and type of fatty acid with which the resins were prepared. On the other hand, ^13^C NMR resonances helped to identify the type of polyalcohol that were used in the synthesis of alkyd resins, by coupling carbon atoms with oxygen atoms in the δ = 40–70 ppm region.

The 2D NMR spectroscopy facilitated the interpretation of chemical resonance assignments of the resin components. The HMQC 2D NMR spectroscopy was used for the identification of ^13^C/^1^H resonance couples, especially for the identification of methylene carbons/proton cross correlations of the fatty acid chain moieties of sacha inchi oil-based alkyd resins. With this, we can compare the degree of unsaturation of the fatty acids chains present in the alkyd resins, enabling the prediction of which resin would have better film performance properties (e.g., drying, hardness, chemical resistance).

GPC results revealed that the molecular weight averages of sacha inchi-based resins increase considerably with more PE content. On the contrary, resins prepared with linseed oil generally decreased their weight-average molar mass by containing more PE. GPC analysis confirmed that dispersity of almost all alkyds was broad. The addition of PE reduced the dispersity values of resins possibly due to the high branching generated by the polyalcohol. Alkyds based on pure glycerol may have broader molecular weight distributions because of the possible formation of side reactions during the monoglyceride process. It was also corroborated that the fatty acid manufacturing process allows synthesizing alkyd resins with a more homogeneous molecular distribution, regardless of their viscosity. Alkyd resins with similar chain lengths can be obtained by working with PE.

## Figures and Tables

**Table 1:** Composition of alkyd resins. Letter “x” indicates the fatty acid source used.

Sample Code	Source	Polyol Ratio (GC:PE)	Fatty Acid Monoglyceride (FA)	Triglyceride, Oil (A)
AS1	sacha inchi	1:0		x
AS2	sacha inchi	0.5:0.5		x
AS3	sacha inchi	0.2:0.8		x
AL1	linseed	1:0		x
AL2	linseed	0.5:0.5		x
AL3	linseed	0.2:0.8		x
FAS1	sacha inchi	1:0	x	
FAS2	sacha inchi	0.5:0.5	x	
FAS3	sacha inchi	0.2:0.8	x	
FAL1	linseed	1:0	x	
FAL2	linseed	0.5:0.5	x	
FAL3	linseed	0.2:0.8	x	

**Table 2:** ^1^H NMR peaks identified (Figure 1) on alkyd resins. The resonances were measured in deuterated chloroform solution and assigned to the polymer structure units [21,29,31,33,34].

Peak	^1^H Chemical Shift Range (ppm)					Group
	FAS3	AS3	AL3	AS1	AL1	
M	7.91–7.63	7.88–7.63	7.97–7.64	7.96–7.65	7.99–7.65	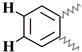
L	7.62–7.37	7.61–7.37	7.61–7.36	7.63–7.38	7.65–7.38	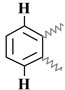
K	5.73–5.21	5.59–5.23	5.80–5.18	5.75–5.18	5.86–5.21	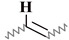
J	4.85–4.28	4.70–4.31	4.74–4.28	4.81–4.28	4.77–4.28	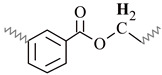
I	4.28–4.04	4.29–4.08	4.27–4.04	4.24–4.07	4.26–4.07	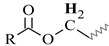
H	3.88–3.39	3.83–3.47	3.86–3.34	3.99–3.46	3.93–3.51	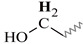
G	2.94–2.63	2.96–2.70	2.94–2.68	2.92–2.66	2.89–2.65	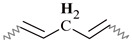
F	2.41–2.21	2.43–2.23	2.47–2.22	2.50–2.23	2.43–2.23	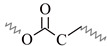
E	2.21–1.87	2.19–1.96	2.19–1.91	2.23–1.89	2.18–1.88	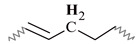
D	1.70–1.50	1.72–1.51	1.74–1.52	1.73–1.52	1.74–1.50	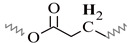
C	1.50–1.12	1.49–1.17	1.49–1.17	1.48–1.18	1.47–1.16	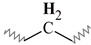
B	1.08–0.94	1.07–0.95	1.06–0.95	1.05–0.95	1.06–0.95	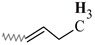
A	0.96–0.80	0.95–0.84	0.94–0.83	0.94–0.82	0.94–0.85	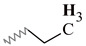

**Table 3:** Molecular weight averages and molecular weight distribution of oil-based alkyd resins.

Sample	${\bar{M}}_{n}$ (10^4^ g/mol)	${\bar{M}}_{w}$ (10^4^ g/mol)	Dispersity (Đ)
AS1	3.4	10.2	3.0
AS2	5.1	11.3	2.2
AS3	12.5	32.4	2.6
AL1	2.5	11.9	4.9
AL2	3.9	7.9	2.0
AL3	3.1	5.3	1.7
FAS1	2.6	8.4	3.2
FAS2	3.1	12.9	4.2
FAS3	8	19.9	2.5
FAL1	26.9	34.7	1.3
FAL2	2.7	7.9	2.9
FAL3	4.1	9.8	2.4

## Data Availability

The raw/processed data required to reproduce these findings cannot be shared at this time as the data also forms part of an ongoing study.
